# Taste perception and oral microbiota are associated with obesity in children and adolescents

**DOI:** 10.1371/journal.pone.0221656

**Published:** 2019-09-11

**Authors:** Chiara Mameli, Camilla Cattaneo, Simona Panelli, Francesco Comandatore, Arianna Sangiorgio, Giorgio Bedogni, Claudio Bandi, Gianvincenzo Zuccotti, Ella Pagliarini

**Affiliations:** 1 Department of Pediatrics, V. Buzzi Children's Hospital, University of Milan, Milan, Italy; 2 Department of Food, Environmental and Nutritional Sciences (DeFENS), University of Milan, Milan, Italy; 3 Pediatric Clinical Research Center “Invernizzi”, University of Milan, Milan, Italy; 4 Clinical Epidemiology Unit, Liver Research Center, Basovizza, Trieste, Italy; 5 Department of Biosciences and Pediatric Clinical Research Center “Invernizzi”, University of Milan, Milan, Italy; Cincinnati Children's, UNITED STATES

## Abstract

Obesity in childhood and adolescence is considered the most prevalent nutritional disorder, in which eating behaviours represent one important factors of influence. Many aspects influence eating behaviours, but taste is considered the main predictor. However, data concerning correlations of obesity, taste sensitivity and behavioural attitudes, such as food neophobia, in children and adolescents are inconsistent. Moreover, it has been suggested that oral bacteria could have a possible role in obesity development and, also, in taste perception. In this context, the present study focused on host related factors with a proposed link to weight gain. To this purpose, taste sensitivity, salivary microbiota composition and food neophobia were compared between children and adolescents with and without obesity in a cross-sectional study. Results showed that children with obesity presented a significantly lower ability in correctly identifying taste qualities and were characterized by a lesser number of Fungiform Papillae (reported as FP/cm^2^) compared to normal-weight subjects. Differences in the ecological indexes of microbial alpha-diversity was found between subjects with obesity and normal-weight ones. Moreover, independently from nutritional status, some bacterial genera seemed to differ between subjects with different sensitivity. The potentiality of this multidisciplinary approach could help to better understand and deepen the sensory-driven and microbiological factors related to weight gain.

## Introduction

Obesity is one of the most serious international health concerns. Prevalence of childhood obesity is increasing worldwide and its rates in Italy are among the highest (36% for boys and 34% for girls) [1,2]. Obesity is considered a multifactorial aetiology disease, which seems to be genetically based, but requires environmental, psychological and social influences to exhibit [3]. An important portion of such environmental influences is represented by diet and related eating behaviours [4]. Although many factors contribute to eating behaviours, taste is considered one of the main predictor in determining children’s food acceptance and choices [5].

It is well known that sensitivity for taste qualities differs between individuals and polymorphisms of the genes coding for taste are supposed to be one of the multifactor causes of these inter-individual differences (for a review see: [6]). Moreover, many researchers reported differences in taste sensitivity between obese and non-obese adults [7–11] as well as children [12,13]. In particular, individuals with a higher body mass index (BMI) are characterized by lower taste sensitivity for all the basic taste and were significantly less responsive to the bitterness of the 6-n-propylthiuracil (PROP) compound, which is considered a phenotypic marker of genetic variation in taste and the most studied one. Consequently, obese subjects need to consume more to have the same stimulation of taste and oral somatosensory system in order to compensate their impaired sensitivity. This lacking sensitivity is hypothesised to have relationships with food intake and body weight variation with implication on long-term health outcomes [14].

However, data concerning correlations between taste sensitivity and obesity are inconsistent [15] and centred mainly on the PROP responsiveness, whereas little is currently known about other taste qualities, especially in children.

Besides individual variation in chemosensory perception, food neophobia (literally the reluctance to eat novel foods) is another aspect to be considered as an important trait in shaping food habits [16]. It has been argued that increased food neophobia may lead children to limit their food choices largely to palatable, high in calories-fat-sugars foods [17], which in turn could represent a risk for excess weight gain. However, studies that have systematically examined the relationship between food neophobia, taste perception and children weight status are scarce and still under investigation [18].

Interestingly, recent research on the enormously complex and vast microbial community in the gastrointestinal tract has provided new insights into the mechanisms of obesity and obesity- related diseases [19]. The majority of studies on the human microbiome focused the attention on the distal gut [20,21], but recently, it has been suggested that oral bacteria could have a potential direct role in development of obesity [22]. However, rather surprisingly, oral microbiota has been poorly investigated in relation to this pathology. Goodson and colleagues [23] reported differences in abundances of salivary bacteria in overweight women compared with normal weight women, suggesting that some taxa could be biomarkers for excess adiposity. In addition, a relationship between sensitivity and oral bacteria was proposed, associating taste perception with the growth of specific oral bacteria [24–27]. Since the composition of oral microbiota appears to have an important but still unclear role in obesity development and to affect sensitivity, an approach to inquiry into the relationship between obesity, taste sensitivity and oral microbiota composition seems required.

In this context, the aim of the present study was to focus on host related factors with a proposed link to weight gain. To this purpose, taste sensitivity, salivary microbiota composition and food neophobia were compared between children and adolescents with and without obesity in a cross-sectional study.

## Material and methods

### Subjects

Participants were recruited at the Obesity Clinic of the V. Buzzi Children’s Hospital (Milan, Italy) from January 31, 2018 to May 31, 2018. The inclusion criteria were: essential obesity with body mass index (BMI) ≥2 standard deviations (SD) according to WHO charts [28], age ≥6 and ≤14 years and Caucasian ethnic group. The exclusion criteria were genetic/syndromic obesity and history of any psychiatric diseases diagnosed according to Diagnostic and Statistical Manual of Mental Disorders (DSMV) [28]. Moreover, we excluded patients with acute or chronic diseases disturbing smell or taste function, with diseases affecting weight or those treated with medications affecting weight (e.g. corticosteroids), patients taking drugs that are known to affect smell or taste and subjects who consumed any antibiotics two months before the study. Healthy sex- and aged-matched controls were recruited as control group from other departments of the clinic applying the same exclusion criteria.

Informed consent has been obtained from all subjects’ parents and/or legal guardians. The study was approved by the Ethics Committee of ASST-FBF-Sacco (Milan, Italy), conducted in accordance with the Declaration of Helsinki and all methods were performed in accordance with the relevant guidelines and regulations.

Each subject was subjected to the anthropometric evaluation, to the collection of saliva samples and to the screening of gustatory functions as well as the evaluation of his/her attitude and preferences towards foods, as described in detail below.

### General procedure

Participants were asked not to eat, to drink nothing but water and not to chew chewing gum at least 2 hours before testing. Participants were subjected to 4 successive sessions. Session 1 included a medical exploration, in which children and adolescents were screened by the medical team and measured for their height and weight to identify the condition of normal-weight or obesity. During Session 2 the oral samplings of saliva were collected. The Session 3 was devoted to the assessment of taste sensitivity (Gustatory function screening and Fungiform Papillae count). During the Session 4, children and adolescents were asked to complete a questionnaire concerning Food Neophobia.

### Anthropometric measurements

Body weight was measured using a medical-certified scale (SECA, Hamburg, Germany) to the nearest 0.1 kg. Height was measured using a children's medical-certified stadiometer (SECA, Hamburg, Germany). BMI was calculated as body mass (W, kg) divided by height (H, m) squared. The BMI values were transformed into BMI z scores using WHO reference values for paediatric BMI [29]. Obesity was defined by BMI z score ≥2 SD (i.e., at least 2 standard deviations above the age- and sex-specific expected value) and normal-weight was defined by BMI between– 2 and 1 SD, in accordance to using WHO reference values for paediatric BMI [29].

### Oral sample collection and DNA Extraction

Subjects were restricted for at least 2 hours (h) of food intake prior to sample collection as mentioned previously. Unstimulated whole saliva samples were collected by direct spitting into a sterile plastic tube in a time span not exceeding 10 minutes (min). Samples were immediately frozen until analysis.

DNA was extracted from 1 ml saliva using the QIAamp DNA Blood Mini Kit, Qiagen (Hilden, DE) and following the protocol suggested by the manufacturer to assure an unbiased representation of bacterial taxa [30]. The DNA concentration of extracted samples was assessed fluorometrically.

### PCR production of 16S rRNA amplicons (V3-V4 regions) and sequencing

For amplicon production, the V3-V4 hypervariable regions of the prokariotic 16S rRNA gene were targeted [31]. PCR was performed in a 50-ml volume containing template DNA, 1x HiFi HotStart Ready Mix (Kapa Biosystems, Wilmington, MA), 0.5 mM of each primer. The cycling program, performed on a Bio-Rad T100 thermal cycler (Bio-Rad, Hercules, CA) included an initial denaturation (95°C for 3 min), followed by 30 cycles at 94°C for 30 seconds (s), 55°C for 30 s, 72°C for 30 s, and a final extension (72°C for 5 min). Clean-up of amplicons was performed using Agencourt AMPure XP SPRI magnetic beads (ThermoFisher Scientific). Illumina sequencing libraries were finally constructed through the link of indexes (Nextera XT Index Kit, Illumina, San Diego, CA), quantified using a Qubit 2.0 Fluorometer (ThermoFisher Scientific, Waltham, MA), normalized and pooled. Libraries were subjected to paired-end sequencing (2 x 300 bp format) on an Illumina MiSeq platform at BMR Genomics (Padova, Italy). Two amplicons were produced and sequenced for each subject enrolled in the study.

### Bioinformatics and community analyses

The bioinformatic treatment of sequencing data was based on the Mothur software [32]. Briefly, raw FASTQ files were quality-filtered using Trimmomatic [33]. High-quality reads were then analysed following the SOP mothur procedure [32]. Chimeric sequences were identified using UCHIME [34] and then removed. The selected sequenced were clustered into operational taxonomic units (OTUs) at 97% similarity using VSEARCH [35]. OTUs were finally annotated, and taxonomy was assigned, against the reference database SILVA [36].

The main ecological indexes of a-diversity Shannon and Chao were computed using Mothur [32]. Diversity in composition among samples (b-diversity) was evaluated at all taxonomic ranks (from phyla to genera) by plotting the relative heatmap using the function heatmap.2 of the Gplots [37] R library, and the relative Principal Component Analysis (PCA) using the R library Ade4 [38].

### Gustatory function screening

The protocol used is fully described elsewhere [13,39]. Gustatory screening was performed applying the ‘Taste Strips’ method [40,41], in which prefabricated filter papers impregnated with different taste solutions were used. The ‘Taste Strips’ method is reported to have a good test-retest-reliability [40], a good acceptance by children and adolescents and has been applied in several research and clinical contexts [41–44]. According to previous studies [13,39,45,46] a total number of 18 paper strips were used, four different concentrations for each taste qualities (sweet, sour, salty and bitter) and two blank strips. The taste strips were presented in increasing concentrations, randomising the taste quality order at each level of concentration. Taste strips were placed on the tongue and subjects were asked to identify the taste quality and to select one of five possible answers (sweet, sour, salty, bitter, no taste) on a form. Before the session started, taste qualities were explained to the participants. In order to control for carry-over effect subjects were asked to rinse their mouth with water before assessment of each taste strip.

### Fungiform Papillae count assessment

The protocol used is fully described elsewhere [9]. The fungiform papillae (FP) count was measured according to Nachtsheim and Schlich [47]. Testing was performed in a sitting position and starting with cleansing the mouth by a sip of water. The child placed the elbows on the table and fixed the head with the hands. The tongue was dried with filter paper and stained with a blue food colorant (F.lli Rebecchi, Color Dolci). A circle of filter paper of 6 mm diameter was used as a template and placed on the left side of the tongue, approximately 1–2 cm from the tip. Several photos of the tongue were taken using a 16-megapixel digital camera (NIKON Corporation, Japan) in macro mode with no flash. After selecting the best photo Adobe Photoshop software was used and three circles were drawn in the front of the anterior tongue using the template. The number of FP was counted inside each marked circles, according to Bakke and Vickers [48] and was counted twice by two independent examiners, and therefore the mean of the two counts was calculated.

### Food Neophobia assessment

To investigate Food Neophobia, participants received the Italian Children Food Neophobia Scale (ICFNS), validated by Laureati and colleagues [49] in a large cohort of school-aged children. The ICFNS consists of eight items, four related to neophobic and four related to neophilic attitudes. In order to aid younger subjects to better understand the level of agreement/disagreement for each item a facial expression is used to exemplify the 5-point scale (‘very false to me’–‘very true for me’). This resulted in a food neophobia score ranging from 8 to 40, which was calculated for each child (neophilic item scores were reversed). Higher scores denote greater food neophobia.

### Statistical analysis

Coarsened exact matching (CEM) was used to match cases with obesity and non-obese controls on the basis of sex (same) and age (within 2 years) [50]. Most continuous variables were not Gaussian-distributed and are all reported as 50th percentile (median) and 25th and 75th percentiles (interquartile range, IQR). Discrete variables are reported as the number and proportion of subjects with the characteristic of interest. Descriptive statistics took CEM into account by means of CEM-related weights [51].

To answer the main study question, i.e. whether there is any difference in gustatory functions between controls and cases, all correctly identified taste strips of the qualities sweet, sour, salty, and bitter were summarised in a Total Taste Score (TTS) giving a maximum score of 16 points. Differences between controls and cases was evaluated by means of a linear regression model (LRM) using TTS as outcome and obesity (0 = no; 1 = yes) as predictor. The LRM took CEM into account by using CEM-related weights and robust 95% confidence intervals [51]. Age and gender were then added to the LRM as covariables to evaluate their potential confounding effect on the relationship between TTS and nutritional status.

The secondary study question, i.e. whether there is a difference in the density of FP between controls and cases, was tested by means of a Poisson regression model (PRM) using the density of fungiform papillae as outcome and nutritional status (0 = control; 1 = case) as predictor. The PRM took CEM into account by using CEM-related weights and robust 95% confidence intervals [51]. Age and gender were then added to the PRM as covariables to evaluate their potential confounding effect on the relationship between FP density and nutritional status.

The third study question involved the characterization of the salivary microbiota composition in cases and controls. This point, descriptive in nature, was addressed at the taxonomic level of phyla and classes by plotting distributions of cases and controls on dot charts [52].

Statistical analysis was performed using Stata 15.1 (Stata Corporation, College Station, TX) together with the user-written CEM command [51].

## Results

From January 31st, 2018 to May 31st, 2018 we recruited 34 subjects affected by obesity and 33 controls, who fulfilled the inclusion criteria. Because of CEM, 45% of the children were female among controls (n = 15) and 56% among cases (n = 19). The median (IQR) age was the same in controls and cases again as an effect of CEM.

The characteristic of cases and controls are detailed in Table 1.

**Table 1:** Characteristics of cases and controls.

	Controls (n = 33)			Cases (n = 34)		
	P_50_	P_25_	P_75_	P_50_	P_25_	P_75_
*Anthropometric measurements*						
Age (years)	10	8	12	10	8	12
Weight (kg)	31.7	27.0	44.0	53.8	40.5	65.7
Weight (SD WHO)*	0.20	-0.24	0.87	2.88	1.90	3.48
Height (m)	1.40	1.27	1.58	1.50	1.33	1.60
Height (SD WHO)	0.42	-0.72	1.05	1.12	0.52	2.29
BMI (kg/m^2^)	17.5	15.3	17.9	24.2	21.2	26.3
BMI (SD WHO)	-0.16	-0.54	0.66	2.30	2.02	2.68
*Gustatory functions*						
Total Taste Score	14	12	15	12	9	13
Sweet taste score	4	3	4	4	3	4
Sour taste score	3	3	3	2	2	3
Salted taste score	4	3	4	3	2	4
Bitter taste score	4	3	4	3	2	4
Fungiform papillae (n/cm^2^)	26	21	28	18	14	22
Neophobia	20	20	26	19	16	24

*Available for children up to 10 years of age.

BMI: body mass index, number; P: percentile; SD: standard deviation; WHO: world health organization

Because of CEM, 45% of the children were female among controls (n = 15) and 56% among cases (n = 19). The median (IQR) age was the same in controls and cases again as an effect of CEM.

### Difference in gustatory functions between cases and controls

A Total Taste Score (TTS) of 16 was the possible maximum score achieved by subjects, obtained by calculating the sum of all four taste qualities presented in the four different concentrations. The correct answers given by subjects in identifying the two blank strips (no taste) have been not been considered for the calculation of the TTS. The TTS obtained in the present study ranged between 5 and 16. As expected, sweet and salty strips were the most often correctly identified, while bitter was the most difficult taste quality to recognize. The mean difference in TTS between cases and controls was -2.3 (95% CI -3.2 to -1.4, p < 0.001, LRM). Such effect size was virtually unmodified after correction for age and gender (mean = -2.4, 95% CI -3.2 to -1.6, p < 0.001, LRM). In general, cases presented significantly more difficulties in correctly identifying the different taste qualities compared to controls, resulting in a lower TTS. Moreover, when considering taste qualities separately, some of them were identified less often by cases. Indeed, the mean difference in sweet score was -0.4 (95% CI -0.7 to 0.0, p < 0.05, LRM), in sour score was -0.7 (95%CI -1.0 to -0.3, p < 0.001, LRM) and in bitter score was -0.7 (95% CI -1.2 to -0.2, p < 0.05, LRM). The components of the main outcome (TTS) are reported for descriptive purposes.

The mean difference in the density of fungiform papillae between cases and controls was -6 FP/cm^2^ (95% CI -8 to -4, p < 0.001, PRM). Such effect size was unmodified after correction for age and gender. In general, controls showed a greater FP density compared to cases.

**Fig 1:**
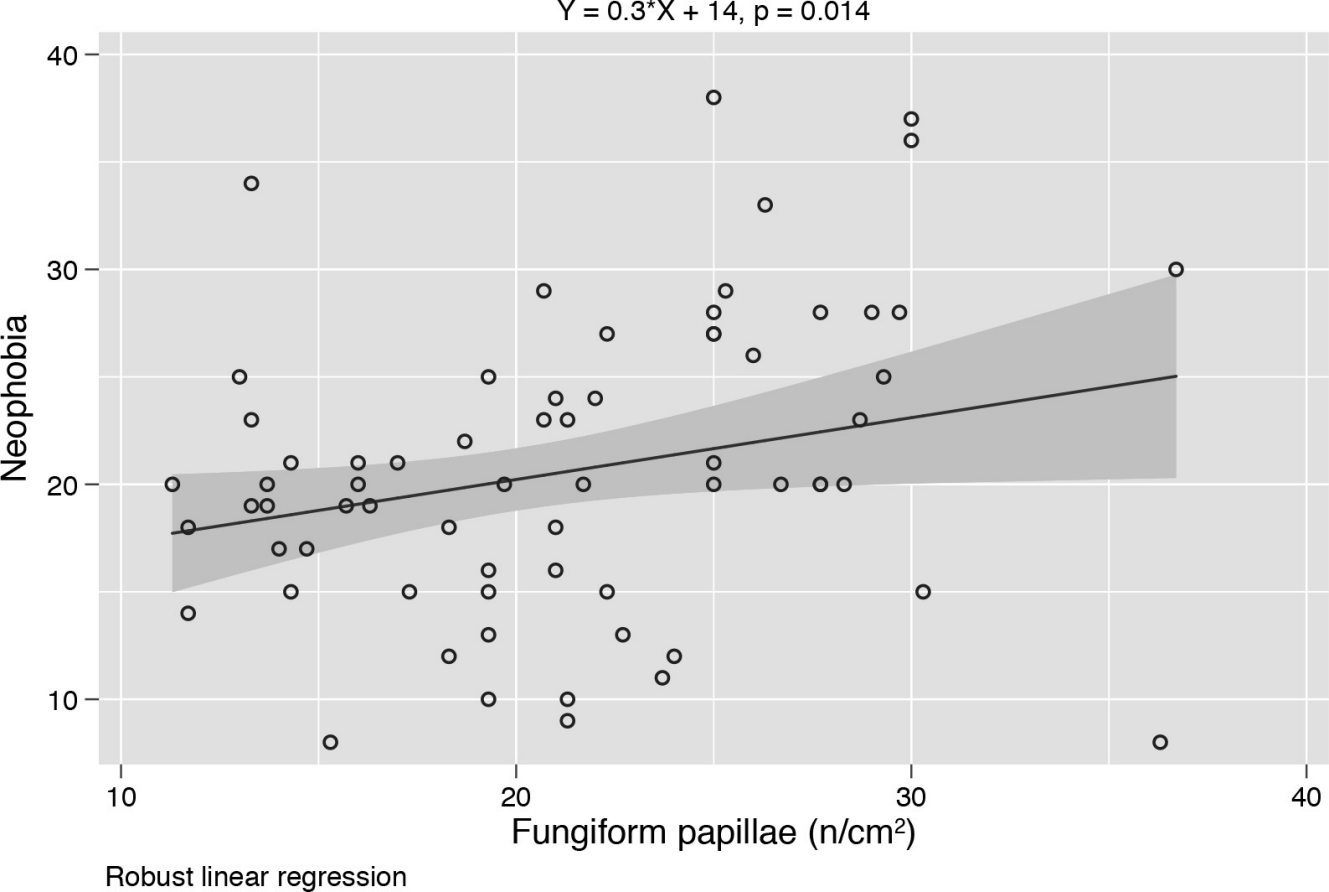
Association between Food neophobia and Fungiform Papillae density. Regression line representing the association between Food neophobia and FP density in the whole sample.

The regression lines representing the neophobia = f(fungiform papillae) had common intercepts (test for common intercepts) and slopes (test for common slopes) so that a single regression can be used to show the association in the whole sample (Fig 1).

#### Difference in oral microbiota composition between cases and controls

Two 16S rRNA amplicons were obtained, sequenced and analysed for each subject. After quality filtering, a total of 11,384,103 high-quality reads were obtained and classified into a total of 76,163 OTUs at 97% similarity level, representing 17 phyla, 32 classes, 61 orders, 120 familiae and 252 genera. The average number of OTUs per sample was 576.9, ranging from a minimum of 322 to a maximum of 1133 (S1 Table).

**Fig 2:**
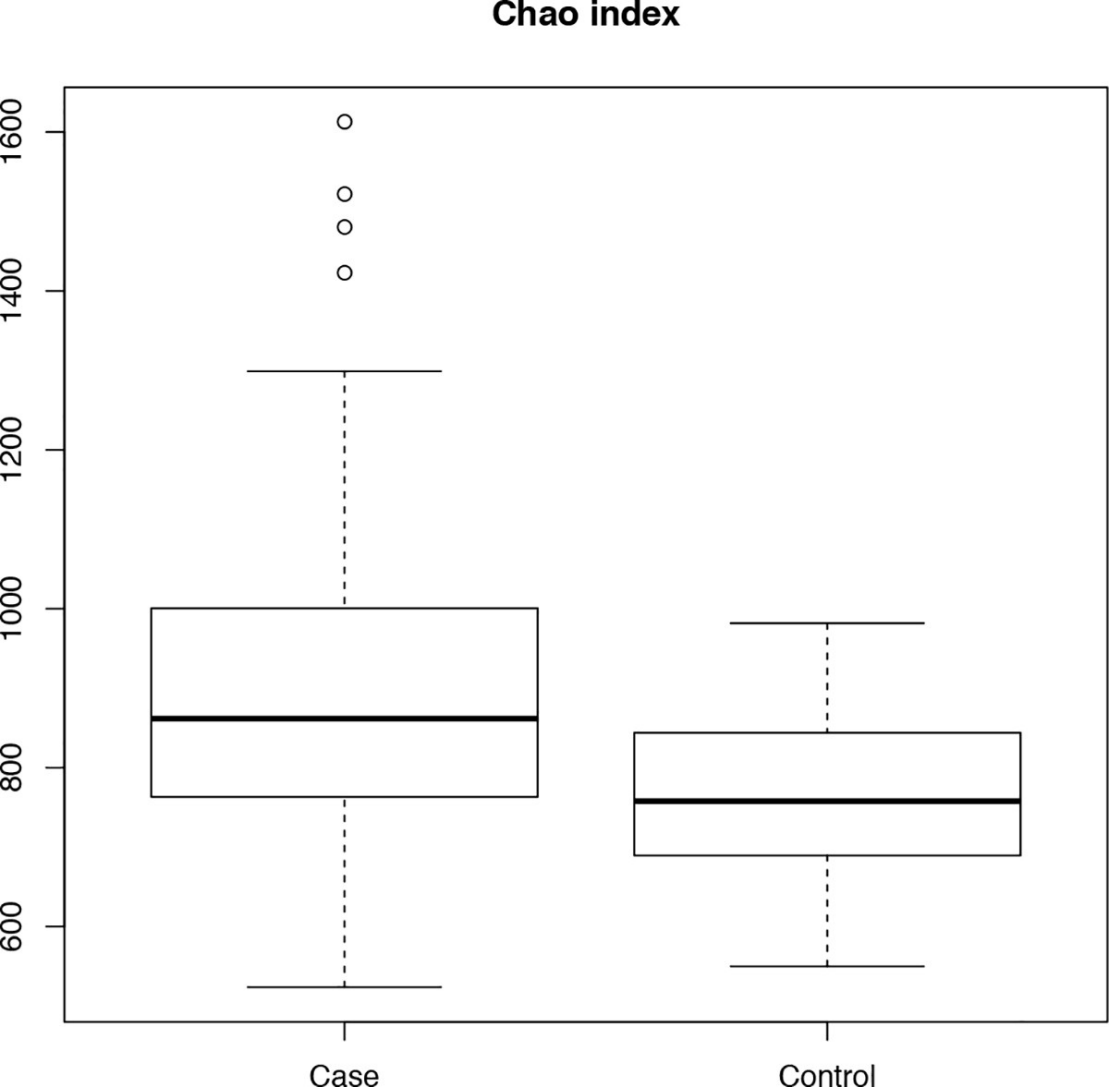
α-diversity (observed species) of the saliva microbiota composition. Box-plots representing the α-diversity (observed species) of the saliva microbiota composition in case and control groups through Chao index.

The median Chao index measured for case samples resulted significantly higher than in control samples (Wilcoxon test, p-value < 0.05, median in case samples 861.7, in control 757.8) (Fig 2). Moreover, for each sample, a rarefaction curve (or individual sample-based rarefaction curves) was drawn by sequentially computing the number of OTUs for an increasing number of reads (S1 Fig).

**Fig 3:**
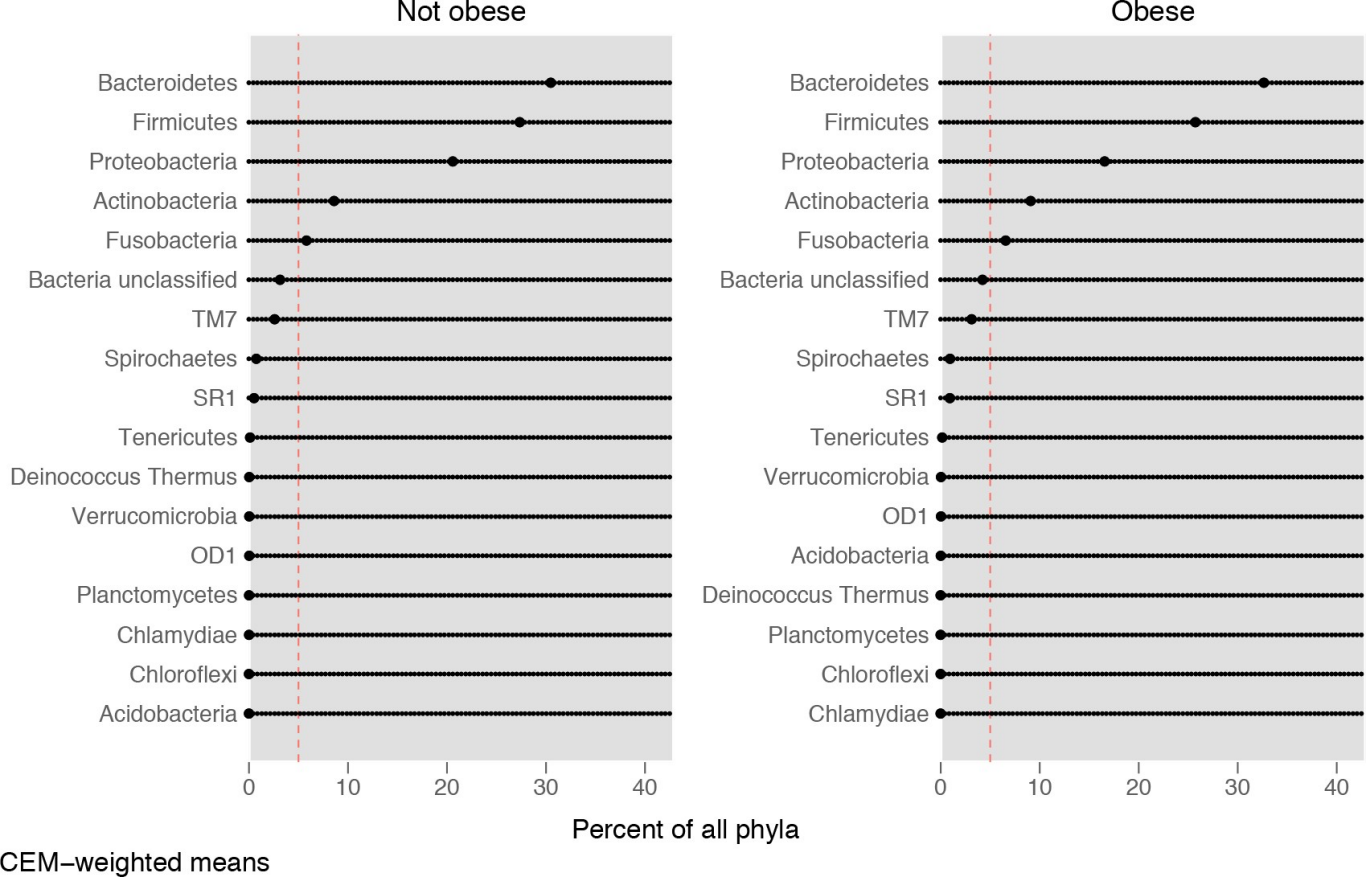
Distribution of the salivary microbiota at the phylum level. Dot chart of the distribution of the salivary microbiota at the phylum level in cases and controls.

The dot chart of the distribution of the salivary microbiota at the phylum level in cases and controls is displayed in Fig 3. The means plotted in the graph are CEM-weighted and, thus, take the case-control matching into account.

The composition at the phylum level resulted very similar in cases and controls. The largest difference was seen for Proteobacteria (22% in controls vs. 17% in cases, Wilcoxon test, p-value < 0.05).

**Fig 4:**
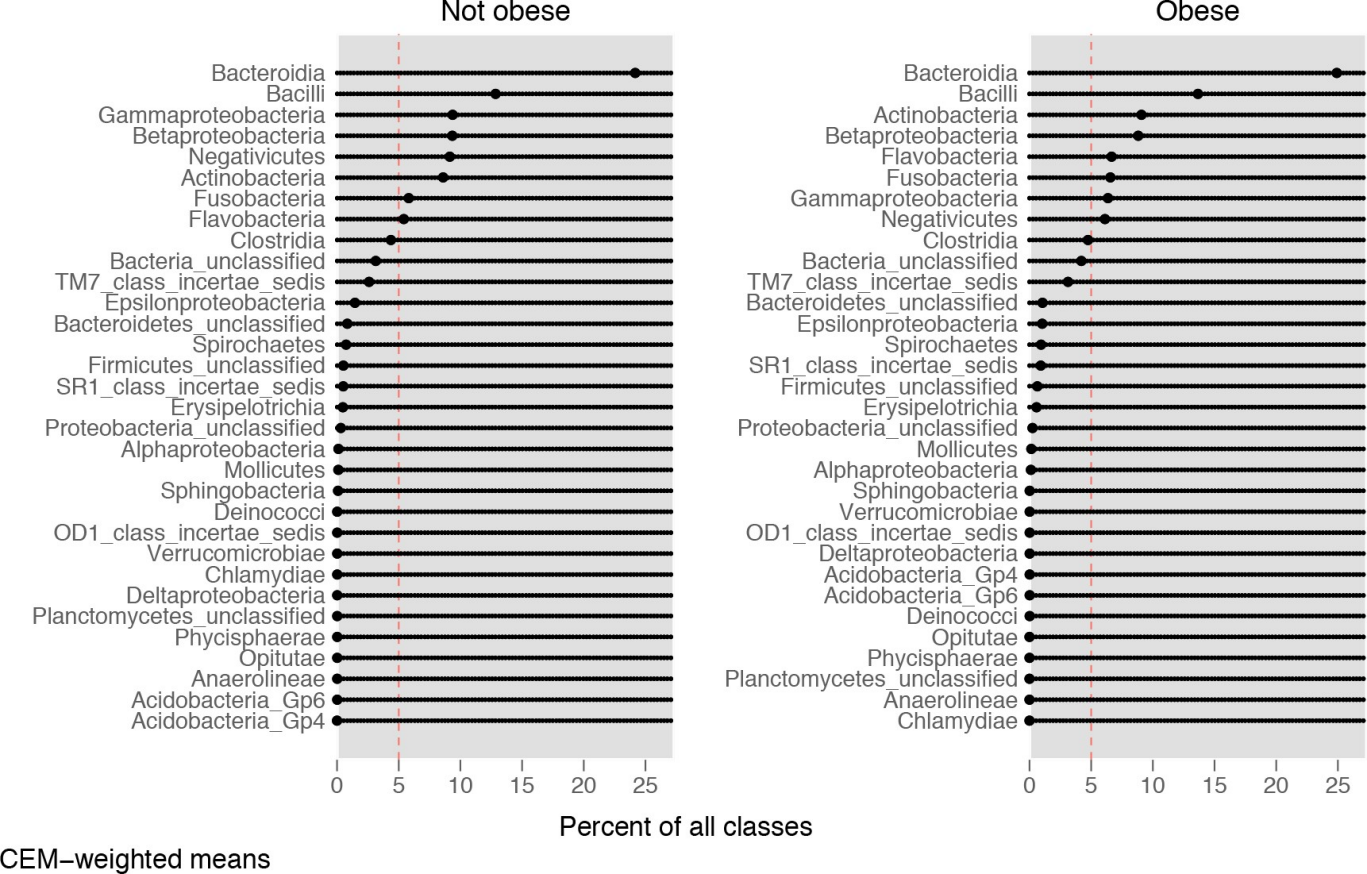
Distribution of the salivary microbiota at the class level. Dot chart of the distribution of the salivary microbiota at the class level in cases and controls.

The composition of the salivary microbiota in cases and controls at the taxonomic level of bacterial classes is reported in Fig 4. The means plotted in the graph are CEM-weighted and, thus, take the case-control matching into account.

Overall, despite minor differences in relative rankings between cases and controls, the composition in bacterial classes resulted similar in cases and controls. The largest difference was observed for Gammaproteobacteria and Negativicutes (9% in controls vs. 6% in cases for both classes).

**Fig 5:**
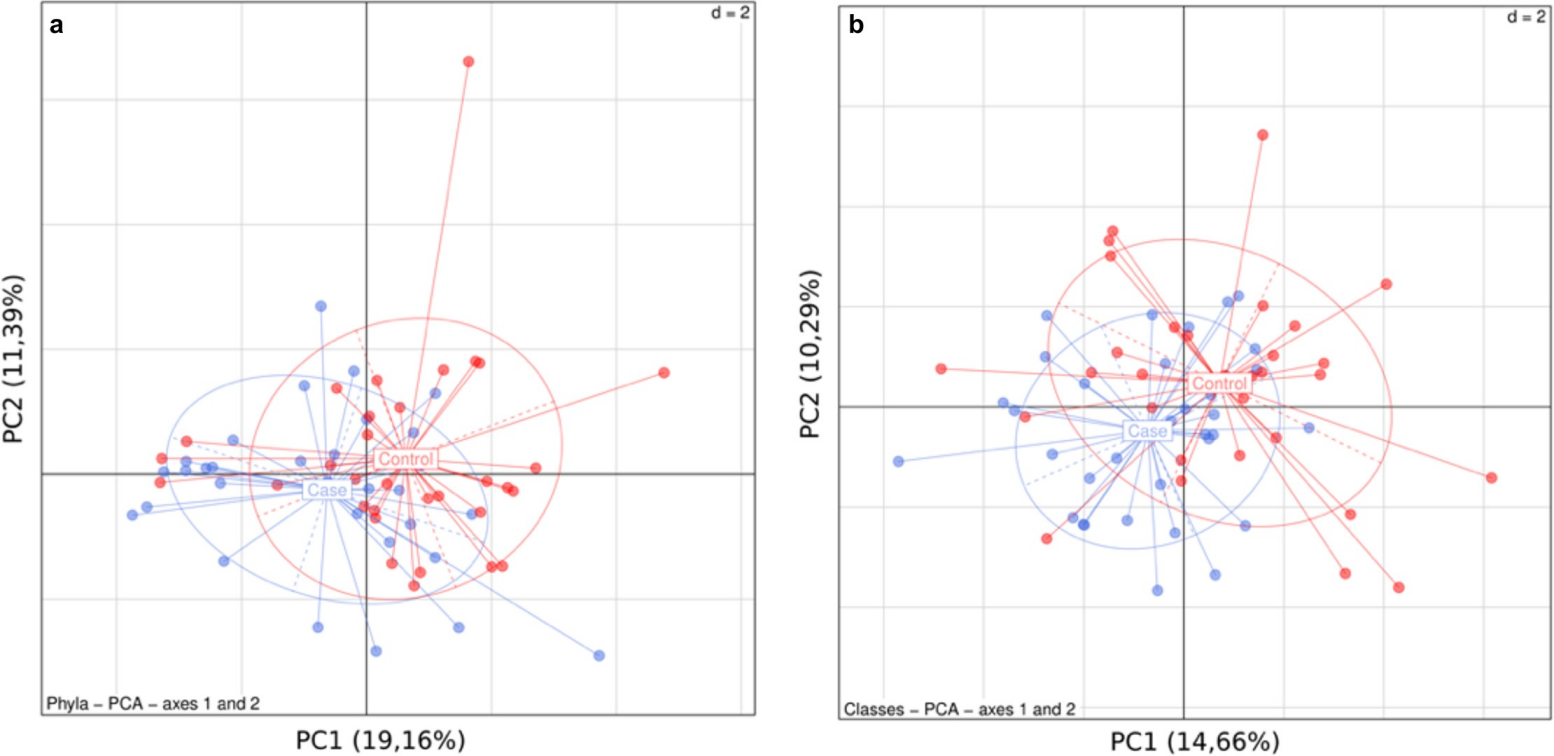
Principal Component Analysis of the microbiota profile. Principal Component Analysis of the microbiota profile in both groups (Controls in red vs Cases in blue) at a) phylum level and at b) class level.

The Principal Component Analysis (Fig 5A and 5B) confirmed that the bacterial consortia presented similar structures in cases and controls, either at phylum (Fig 5A) and class (Fig 5B) taxonomic levels.

**Fig 6:**
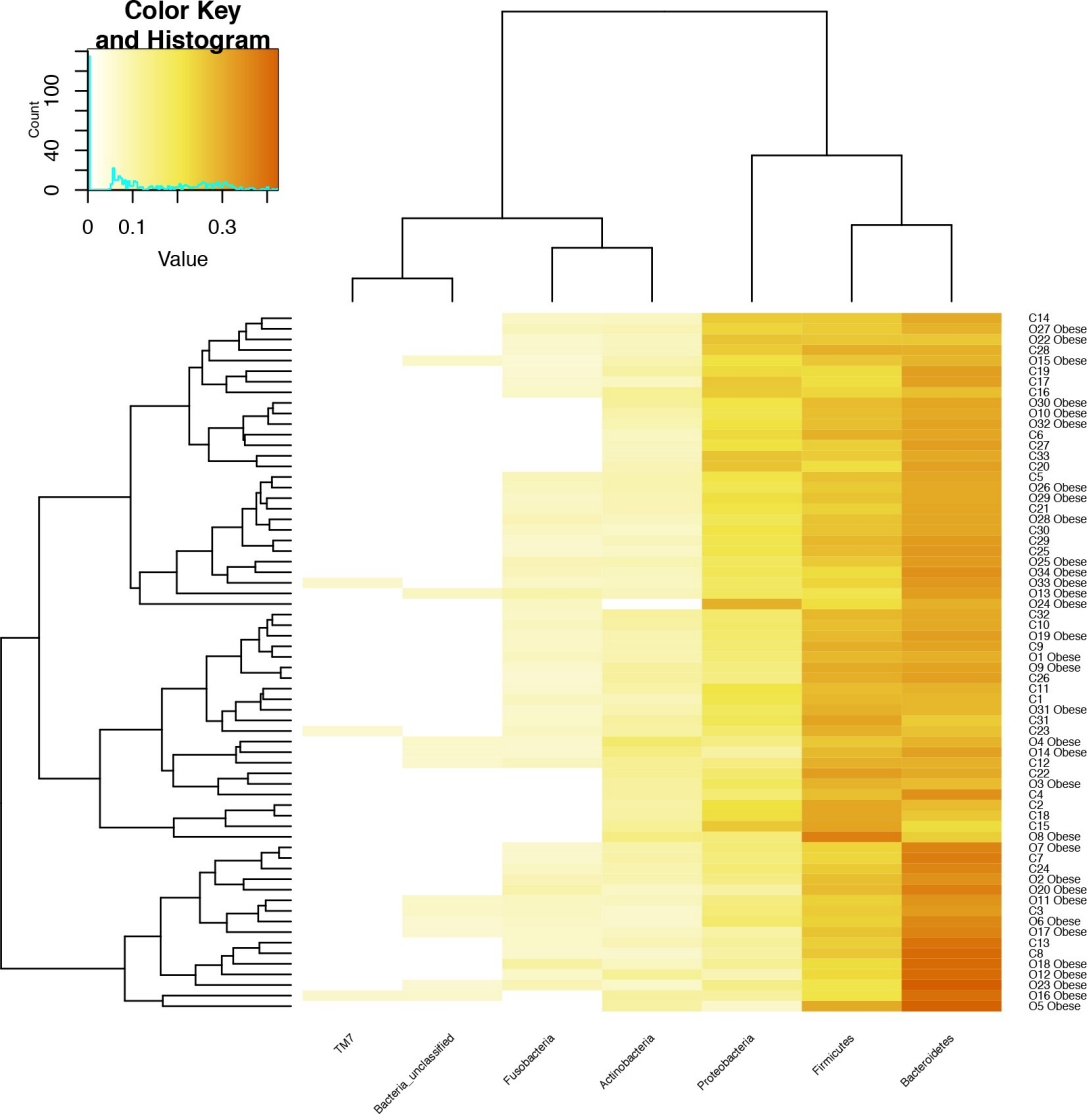
Correlations between the subjects and the abundance levels of selected phyla. Heatmap representing the correlations between the subjects and the abundance levels of selected phyla that were represented in the microbiota samples.

In Fig 6 is shown the heatmap based on the Euclidean distance of the most abundant bacterial phyla and on the dendrogram produced by the clustering analysis.

From this analysis it emerges that subjects do not seem to cluster based on their nutritional status. Instead, a cluster characterized by a higher representation in Bacteroidetes, which comprised either cases and controls, seemed the only one to emerge from this analysis. It seemed, thus, interesting to compare subjects chosen for being at the ‘extremes’ of Bacteroidetes abundance, independently from their nutritional status. Within the study cohort, we selected 7 subjects characterized by the highest Bacteroidetes abundance (dark orange colour in Fig 6) and 7 subjects characterized by the lowest Bacteroidetes abundance (light yellow colour in Fig 6), forming 2 clusters, named respectively ‘Group 1’ and ‘Group 2’, the characteristics of which are given in S2 Table. These groups seem to differ for the ability of subjects in correctly identifying the different taste qualities, and especially the bitter taste. In particular, Group 1 presented a general lower TTS (11.43 ± 3.15 vs 14.00 ± 0.00) and, especially, a lower ability in identifying bitter taste compared to Group 2 (2.43 ± 1.13 vs 4.00 ± 0.00).

## Discussion

It is becoming clear that the origin of obesity is multifactorial disease and the purpose of this study was to deepen the investigation on the host related factors proposed as potential causes affecting weight gain. In recent years, compelling evidence has been accumulated on the relations between taste perception and body mass index, suggesting that individuals with a higher BMI showed reduced taste sensitivity [for a review see 15]. However, the majority of studies have focused on the relation between nutritional status and taste perception in adults. Moreover, bitter sensitivity in relation to BMI was mainly examined for the PROP compound. Data in literature about the sensitivity of children towards all taste qualities appear to be incomplete and, in particular, little is currently known about the perception of other bitter compounds [12–14]. Indeed, only Overberg and colleagues [13] investigated the relationship between taste sensitivity for all five taste qualities and nutritional status in children and adolescents with and without obesity, showing a higher sensitivity for all tastes in the former. Accordingly, the hypothesis that children and adolescents characterized by a different nutritional status, presented differences in their taste sensitivity was confirmed in the present study, with subjects with obesity showing a lower ability in correctly identifying taste qualities compared to the group of controls. Taste sensitivity has also been evaluated by measuring and counting the number of FP/cm^2^. Because FPs contain the taste buds of the anterior tongue, many literature data suggest that individual differences in their density and size (e.g. diameter) could be responsible of different chemosensory perception among individuals [53–55]. Moreover, a negative correlation between FP density and obesity was suggested in adults [9,10]. Our findings seem to be in agreement with this hypothesis, showing that normal-weight controls presented a greater density of FP and were also more sensitive to basic tastes than subjects with obesity. This impaired taste perception in children and adolescents with obesity supports the assumption that the taste system is impaired in subjects affected by this disease [9,10–13]. We can presume that, as a results of low taste sensitivity, high amounts of tastants would be required to elicit a response within taste receptor cells, which in turn may affect eating behaviour, contributing to excess energy intake and perhaps increasing obesity.

As previously reported, obesity is considered a disease with a multifactor aetiology, thus, other factors not strictly related to taste perception could be involved in weight gaining. Indeed, previous studies showed that body weight could be associated with some traits related to personality, such as food neophobia [9,18,56]. Quite surprisingly, there has been very little research carried out to ascertain the relationship between food neophobia, taste perception and nutritional status. Food neophobia is considered a maladaptive behaviour, which can lead to decreased dietary variety and quality. Food neophobics may choose to eat familiar food, normally more energy-dense than healthier food, which could clearly affect their nutritional status leading them to a greater prevalence of overweight [10].

However, our results did not highlight any relationship between nutritional status and food neophobia, accordingly to previous studies already conducted with children [49] and young adults [57]. Concerning about the relationship between food neophobia and gustatory functions, the present study showed that, independently of nutritional status, children and adolescents who present an higher FP density are significantly more neophobic than less sensitive individuals, suggesting that neophobic reactions could be associated with higher sensitivity. Our results are in agreement with literature data reporting that children, who are more sensitive to taste or tactile sensations, have fewer positive consequences when trying new foods, particularly those characterized by strong sensory properties, leading to greater neophobic attitudes [54,58]. However, it is still unclear whether the food rejection shown by neophobic subjects is facilitated by higher arousal levels when approaching new foods or by an actual physiological predispositions to taste hypersensitivity [59].

In this study, we also focus on the link between oral bacterial community and obesity. The analysis of the salivary bacterial consortia revealed that people with obesity have a higher bacterial richness than normal weight controls. This result is in contrast with the current literature which normally reports decreases in ecological indexes of bacterial richness and diversity as a trademark of many dysbiotic states, characterizing a variety of pathological conditions, among which obesity. Indeed, decreased gut microbiome diversity has been linked to obesity [60,61]. Similar associations between the altered microbial diversity and unhealthy or inflammatory states in the host have been found with the oral microbiota [62,63].

Data in literature reported that significant differences in the gut microbiome has been found between people with obesity and controls [20]. Data from animal models and human studies have shown correlations between alteration in gut phyla and obesity disease, but results are inconsistent [20,22,64]. In the present study, very few significant variations in relative abundances of some taxa were noted between cases and controls at higher taxonomic levels in the salivary microbiome. The lack of greater variation between the salivary microbiota of these groups may be due to the relatively small sample size of each group. It is also possible that the young age of the subjects involved and the relatively shorter duration of their disease do not allow to highlight a clear microbial flora variation. It is notable that past researches on the relationship between the oral microbiota composition and obesity, have yielded contradictory results. For example, it has been reported that levels of many bacteria differed in the saliva of overweight women when compared with healthy individuals [23]. Specifically, these authors found that *Prevotella spp*. (belonging to Bacteroidetes) was more abundant in the overweight while *Selenomonas spp*. was present only in the overweight individuals, suggesting that these taxa could be biomarkers for excess adiposity. Moreover, Ziegler and colleagues [65] suggested an association between obesity and bacterial cellular abundance of Firmicutes and Actinobacteria in oral biofilm. However, recent studies reported no differences in oral microbiota composition according to BMI [26,66].

The role oral microbiota plays in influencing taste perception is a novel field of investigation. Recently, a relationship between taste sensitivity and oral bacteria was suggested, associating taste perception with the growth of specific taxa. Solemdal and colleagues [25] found that sour taste was particularly impaired in children with high *lactobacilli* growth. They suggested that the acids produced by the bacteria may cause an adaption in sour taste perception, thus increasing their sour taste threshold. In addition, non-taster children, who presented a decreased taste sensitivity for PROP, were associated with higher *mutans streptococci* counts [67]. However, these assumptions were not supported by microbiomic or predicted metagenome analyses. Our previous studies, which investigated both taste perception and oral microbiota, applying reliable and sensitive methods and using microbiomic analysis of tongue microbial ecosystem, supported the hypothesis that oral bacteria may have a role in influencing and/or modulating taste perception [24,27]. Indeed, we reported that young adults with a reduced taste responsiveness are characterized by different oral microbiota composition, in agreement with previous findings [26,68]. In the present study, due to the selection of few samples belong to the two groups accordingly to heatmap, we conducted descriptive statistics rather than inductive statistics and no conclusions about associations can be drawn. Interestingly, however, it seems that there is an increase in the proportion of Bacteroidetes and Bacteroidia and a decrease in the proportion of Proteobacteria in Group 1, which includes subjects characterized by a general lower ability to perceive all the taste qualities and, especially bitter taste. In conclusion, the results of this study support the hypothesis that children and adolescents with a different nutritional status differ in their taste sensitivity. However, these cross-sectional results are required to be confirmed through longitudinal studies. No relations were found between nutritional status and food neophobia, however, independently of nutritional status, children and adolescents who present a greater FP density are significantly more neophobic than less sensitive individuals. We report that our obese and normal-weight subjects differ for the ecological indexes of microbial alpha-diversity. Some minor differences in taxa composition were also noticed, (e.g. for Proteobacteria). Moreover, independently from nutritional status, some bacterial genera seemed to differ among subjects with different ability in perceiving taste qualities. Further exploration of the oral microbiome in relation to taste perception and nutritional status will enhance our understanding of the host related factors that are proposed as potential causes affecting weight gain. This multidisciplinary approach offer new insights into the reciprocal impact between host related factors and obesity, and could open new strategy lines for obesity prevention and therapy in childhood.

## Supporting information

S1 Table Number of reads per 35 sample after filtering. (PDF)

S2 Table Characteristics of study participants belong to Group 1 and Group 2. (PDF)

S1 File Supporting data. (XLSX)

S1 Fig Rarefaction curves within samples. (PDF)
