# Supplementary material for: Taste perception and oral microbiota are associated with obesity in children and adolescents
Source: PLoS One. 2019 Sep 11;14(9):e0221656. doi: 10.1371/journal.pone.0221656 (PMC6738620; doi:10.1371/journal.pone.0221656)
Supplement: S1 Table — (PDF) [file pone.0221656.s001.pdf]

S1 Tab. Number of reads per sample after filtering

Sample	nr. of reads
C1_repeat1	92305
C1_repeat2	88168
C10_repeat1	94210
C10_repeat2	84205
C11_repeat1	76577
C11_repeat2	106399
C12_repeat1	102343
C12_repeat2	83016
C13_repeat1	107531
C13_repeat2	102039
C14_repeat1	111308
C14_repeat2	104019
C15_repeat1	108268
C15_repeat2	122074
C16_repeat1	98132
C17_repeat1	96435
C17_repeat2	89789
C18_repeat1	117087
C18_repeat2	96490
C19_repeat1	84574
C19_repeat2	102132
C2_repeat1	96387
C2_repeat2	93567
C20_repeat1	82885
C20_repeat2	113782
C21_repeat1	116695
C21_repeat2	118034
C22_repeat1	126141
C22_repeat2	93869
C23_repeat1	75043
C23_repeat2	73903
C24_repeat1	91381
C24_repeat2	80473
C25_repeat1	76784
C25_repeat2	86707
C26_repeat1	84962
C26_repeat2	94676
C27_repeat2	110746

C28_repeat1	77215
C28_repeat2	74817
C29_repeat1	83194
C29_repeat2	82165
C3_repeat1	80131
C3_repeat2	77578
C30_repeat1	66930
C30_repeat2	84503
C31_repeat1	79761
C31_repeat2	75737
C32_repeat1	86335
C32_repeat2	75615
C33_repeat1	83292
C33_repeat2	88312
C4_repeat1	79941
C4_repeat2	87314
C5_repeat1	67315
C5_repeat2	94438
C6_repeat1	74668
C6_repeat2	76488
C7_repeat1	83148
C7_repeat2	80756
C8_repeat1	103642
C8_repeat2	82985
C9_repeat1	88831
C9_repeat2	75607
OB1_repeat1	114139
OB1_repeat2	92166
OB10_repeat1	89937
OB10_repeat2	55579
OB11_repeat1	69522
OB11_repeat2	71730
OB12_repeat1	79056
OB12_repeat2	119528
OB13_repeat1	66981
OB13_repeat2	75604
OB14_repeat1	88635
OB14_repeat2	41131
OB15_repeat1	46569
OB15_repeat2	67806

OB16_repeat1	34295
OB16_repeat2	90679
OB17_repeat1	58481
OB17_repeat2	68829
OB18_repeat1	82286
OB19_repeat1	79842
OB19_repeat2	84145
OB2_repeat1	95978
OB2_repeat2	56131
OB20_repeat1	109112
OB20_repeat2	103267
OB22_repeat1	91715
OB22_repeat2	89636
OB23_repeat1	103325
OB23_repeat2	108016
OB24_repeat1	88377
OB24_repeat2	95520
OB25_repeat1	97936
OB25_repeat2	78828
OB26_repeat1	89449
OB26_repeat2	81828
OB27_repeat1	94477
OB27_repeat2	79616
OB28_repeat2	86107
OB29_repeat1	93720
OB29_repeat2	86279
OB3_repeat1	103306
OB3_repeat2	95465
OB30_repeat1	122241
OB30_repeat2	90231
OB31_repeat1	87822
OB31_repeat2	82477
OB32_repeat1	69230
OB32_repeat2	86538
OB33_repeat1	73869
OB33_repeat2	89069
OB34_repeat1	87530
OB34_repeat2	73822
OB4_repeat1	118773
OB4_repeat2	103181

OB5_repeat1	97589
OB5_repeat2	135662
OB6_repeat1	111152
OB6_repeat2	53249
OB7_repeat1	102638
OB7_repeat2	122062
OB8_repeat1	106505
OB8_repeat2	65293
OB9_repeat1	85329
OB9_repeat2	116180
