# Supplementary material for: Taste perception and oral microbiota are associated with obesity in children and adolescents
Source: PLoS One. 2019 Sep 11;14(9):e0221656. doi: 10.1371/journal.pone.0221656 (PMC6738620; doi:10.1371/journal.pone.0221656)
Supplement: S2 Table — (PDF) [file pone.0221656.s002.pdf]

S2 Tab. Characteristics of study participants belong to Group 1 and Group 2.

Group	Participant ID	Status	BMI (SD)	Age (SD)	Gender F:M	TTS (SD)	Sweet TS (SD)	Sour TS (SD)	Bitter TS (SD)	Salty TS (SD)
	O 7	Case	21.89 (1.72)	9.57 (2.29)	4:3	11.43 (3.15)	3.29 (0.76)	2.71 (0.76)	2.43 (1.13)	3.00 (1.15)
	C 13	Control								
	O 18	Case								
1	C 8	Control								
	O 12	Case								
	O 5	Case								
	O 23	Case								
	C 15	Control	19.77 (1.19)	9.57 (1.19)	3:4	14.00 (0.82)	3.86 (0.38)	2.53 (0.53)	4.00 (0.00)	3.57 (0.53)
	O 8	Case								
	C 31	Control								
2	C 18	Control								
	O 22	Case								
	C 23	Control								
	C 16	Control								

3
