# Supplementary figures and images for: Taste perception and oral microbiota are associated with obesity in children and adolescents
Source: PLoS One. 2019 Sep 11;14(9):e0221656. doi: 10.1371/journal.pone.0221656 (PMC6738620; doi:10.1371/journal.pone.0221656)

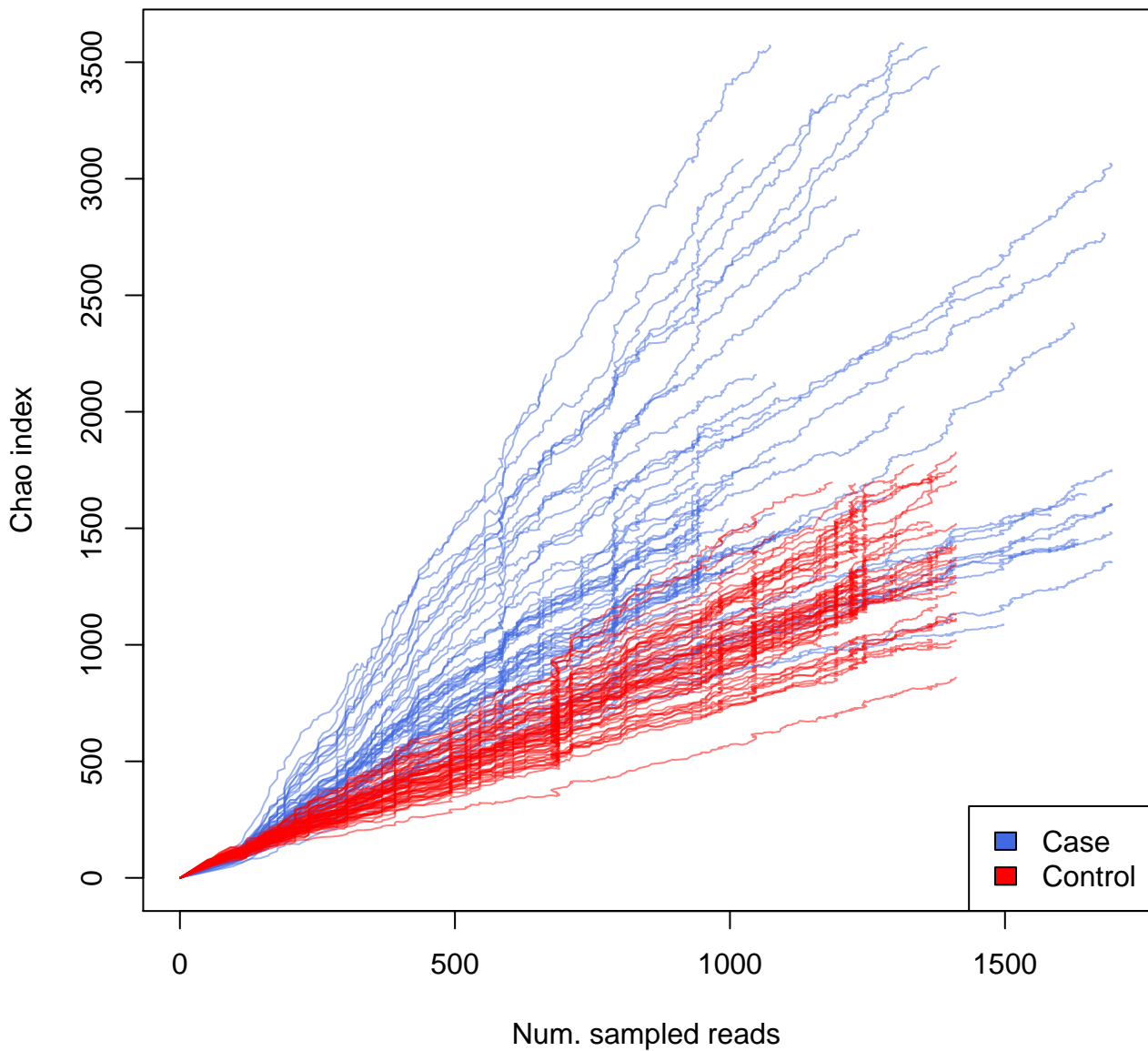

Supplement: S1 Fig — (PDF) [file pone.0221656.s004.pdf]
